# Comparison of Optic Disc Morphology of Optic Nerve Atrophy between Compressive Optic Neuropathy and Glaucomatous Optic Neuropathy

**DOI:** 10.1371/journal.pone.0112403

**Published:** 2014-11-06

**Authors:** Masayuki Hata, Kazuaki Miyamoto, Akio Oishi, Yukiko Makiyama, Norimoto Gotoh, Yugo Kimura, Tadamichi Akagi, Nagahisa Yoshimura

**Affiliations:** Department of Ophthalmology and Visual Sciences, Kyoto University Graduate School of Medicine, Kyoto, Japan; Harvard Medical School, United States of America

## Abstract

**Objectives:**

To compare the optic nerve head (ONH) structure between compressive optic neuropathy (CON) and glaucomatous optic neuropathy (GON), and to determine whether selected ONH quantitative parameters effectively discriminate between GON and CON, especially CON cases presenting with a glaucoma-like disc.

**Methods:**

We prospectively assessed 34 patients with CON, 34 age-matched patients with moderate or severe GON, and 34 age-matched healthy control subjects. The quantitative parameters of ONH structure were compared using the Heidelberg Retina Tomograph 2 (HRT2) and Spectralis optical coherence tomography with an enhanced depth imaging method.

**Results:**

The mean and maximum cup depths of CON were significantly smaller than those with GON (*P*<0.001 and *P*<0.001, respectively). The distance between Bruch's membrane opening and anterior surface of the lamina cribrosa (BMO-anterior LC) of CON was also significantly smaller than that of glaucoma but was similar to that of the healthy group (*P*<0.001 and *P* = 0.47, respectively). Based on Moorfields regression analysis of the glaucoma classification of HRT2, 15 eyes with CON were classified with a glaucoma-like disc. The cup/disc area ratio did not differ between cases of CON with a glaucoma-like disc and cases of GON (*P* = 0.16), but the BMO-anterior LC and mean and maximum cup depths of CON cases with a glaucoma-like disc were smaller than those in GON (*P* = 0.005, *P* = 0.003, and *P* = 0.001, respectively).

**Conclusions:**

Measurements of the cup depths and the LC depth had good ability to differentiate between CON with a glaucoma-like disc and glaucoma. There was no laminar remodeling detected by laminar surface position in the patients with CON compared to those with GON.

## Introduction

Enlargement of optic disc cupping is a classical sign of glaucoma, but it also can result from nonglaucomatous neurological lesions, such as ischemic optic neuropathy, hereditary optic neuropathy, traumatic optic neuropathy, and compressive optic neuropathy (CON) [1]–[6]. Many reports indicate that intracranial lesions often mimic the clinical presentation of glaucoma and result in misdiagnosis [2], [3], [7]–[9]. Detecting CON among eyes with glaucoma and a glaucoma-like disc is critically important because intracranial lesions, including a brain tumor and intracranial aneurysm, are life-threatening and require treatments that are entirely different from that of glaucoma, and the delay in diagnosis or misdiagnosis can be fatal.

Differentiating glaucomatous from nonglaucomatous disc cupping is often difficult. Trobe and associates showed that pallor of the neuroretinal rim is useful in predicting nonglaucomatous cupping in a review of optic nerve head (ONH) photographs, but such funduscopic characteristics are subjective; the degree of pallor is influenced by disturbances of the ocular media and variations in photographic technique, and even experienced observers often misdiagnose the etiology of the cupping [2]. Other clinical findings such as dyscromatopsia or certain visual field characteristics can help differentiate between the two diseases; however, their usefulness is limited. The depth of optic disc cupping is considered one of the most important objective findings of the ONH, which helps to differentiate nonglaucomatous optic neuropathy from glaucoma. Mashima and associates compared the ONH between eyes with glaucoma and those with hereditary optic neuropathy using the Heidelberg Retina Tomograph (HRT) parameters [10]. They reported that 73% of eyes with hereditary optic neuropathy were misdiagnosed with glaucoma, but the mean cup depth and maximum cup depth of eyes with hereditary optic neuropathy were significantly smaller than those of eyes with glaucoma. However, characteristics of cupping depth for eyes with CON remained unknown. The quantitative analysis of optic disc cupping may provide useful data to distinguish CON from glaucoma. Additionally, other ONH structures characteristic of glaucoma, such as lamina cribrosa (LC) degeneration [11]–[13], are candidates for objective parameters for differentiating sight-threatening and life-threatening optic atrophy from glaucoma. However, in vivo evaluation of the LC was not easy.

Recently, a new approach to optical coherence tomography (OCT), known as enhanced depth imaging (EDI) OCT, allows visualization of deeper layers of the ONH, including the LC [14]–[18]. These studies showed that the LC was located posteriorly and significantly thinner in patients with glaucoma than in healthy controls. However, the morphology of these ONH structures in eyes with an enlarged optic cup caused by nonglaucomatous optic neuropathy has not been elucidated. In this prospective study, we compare, for the first time, the ONH quantitative parameters, including cup/disc (C/D) area ratio, cup depth, disc size, LC depth and prelamina tissue thickness (PLT), between CON and glaucomatous optic neuropathy and determine whether these ONH parameters effectively discriminate between eyes with glaucoma and eyes with CON, especially in eyes with CON presenting with a glaucoma-like disc.

## Methods

This prospective, cross-sectional, comparative study was carried out with approval by the Institutional Review Board (IRB) at Kyoto University Graduate School of Medicine, and all studies conducted adhered to the tenets of the Declaration of Helsinki. Written informed consent was obtained from each participant after a detailed explanation of the nature and possible consequences of the study procedures and the IRB approve this consent procedure. Compressive optic neuropathy patients who visited the Neuro-ophthalmology Clinic of Kyoto University Hospital between August 2012 and September 2013 were recruited for this study. Age-matched open-angle glaucoma and healthy control subjects were also recruited during the same study period from the Glaucoma Clinic of the same hospital. Subjects were included only if they fulfilled the eligibility requirements detailed below and signed an informed consent form at the screening visit. When both eyes were eligible, 1 eye was randomly selected for inclusion in the study.

All subjects underwent comprehensive ophthalmic assessment, including visual acuity measurements with a Landort chart, intraocular pressure (IOP) measurement using a Goldmann applanation tonometry, slit-lamp examinations, stereo disc photography with a 3-Dx simultaneous stereo disc camera (Nidek Co., Ltd, Gamagori, Japan), axial length measurement with an IOLMaster biometer (Carl Zeiss Meditec, Inc., Dublin, California, USA), and standard automated perimetry with the Humphrey Visual Field Analyzer using the 24–2 Swedish Interactive Threshold Algorithm (SITA) standard strategy (Carl Zeiss Meditec, Inc., Dublin, CA, USA) within 3 months from the date of OCT. Experienced ophthalmologists performed spectral domain (SD)-OCT (Spectralis; Heidelberg Engineering GmbH, Dossenheim, Germany) and the Heidelberg Retina Tomograph 2 (HRT2; Heidelberg Engineering GmbH, Heidelberg, Germany).

### Inclusion criteria

Inclusion criteria for compressive optic neuropathy subjects included the following: (1) optic nerve atrophy caused by compression of the anterior visual pathway by a brain tumor or aneurysm confirmed by cranial neuroimaging with magnetic resonance imaging, (2) history of surgical treatment for the causative disease more than 1 year before this study, (3) mean deviation (MD) measured with the Humphrey visual field analyzer of −6 dB or less, and (4) IOP of 20 mm Hg or less. Inclusion criteria for open-angle glaucoma subjects were as follows: (1) a clinical diagnosis of open-angle glaucoma with documented progressive optic disc change, such as a vertical cup-to-disc ratio of 0.7 or greater, intraindividual asymmetry of 0.2 or more, or the presence of focal thinning, notching, and disc hemorrhage, and associated glaucomatous loss of visual field; (2) repeatable glaucomatous visual field loss as measured with the Humphrey field analyzer with the standard 24–2 SITA program on at least 2 subsequent tests; (3) treated IOP of 20 mm Hg or less; and (4) MD measured with Humphrey visual field analyzer of -6 dB or less. Inclusion criteria for healthy normal eyes included (1) an IOP of 20 mm Hg or less with no history of increased IOP, (2) normal visual field testing results, and (3) an absence of glaucomatous optic disc appearance on stereo disc photography.

The appearance of the optic disc on stereoscopic photographs was evaluated by glaucoma specialist (YK and TA) who was masked to all other information about the eyes. Determination of the appearance of the optic disc (glaucomatous or normal) was performed in accordance with the assessments of the two examiners. For all subjects in the three groups, a vertical cup-to-disc ratio was measured on stereo disc photography.

### Exclusion criteria

Exclusion criteria were as follows: (1) high myopia defined as less than −6.0 diopters (D); (2) astigmatism more than 3 D; (3) any other ophthalmic disease, including media opacity, diabetic retinopathy, or other diseases affecting the visual fields, such as ischemic optic neuropathy, optic neuritis, uveitis, retinal or choroidal diseases, and trauma; (4) a tilted optic disc, which was defined as an index of tilt (ratio of minimum to maximum optic disc diameter) less than 0.75 on stereo disc photography [19]; and (5) a history of intraocular surgery or laser treatment except for uncomplicated cataract surgery.

Age matching in the glaucoma patients and healthy subjects was performed by randomly selecting one subject within 2 years of the same age for CON.

### Confocal scanning laser tomography

Confocal scanning laser tomography of the optic disc was performed on all participants in this study with HRT2 (Heidelberg Engineering GmbH, Heidelberg, Germany) to evaluate the optic disc size, cup/disc area ratio, and mean and maximum cup depths. Three images were acquired automatically after initial positioning by an experienced operator. Qualified HRT scans were well-centered and well-focused. The ONH contour line was then drawn by the same operator with the ONH margin defined as the inner border of Elschnig's ring, and global stereometric parameters for HRT2 were acquired. Moorfields regression analysis (MRA) categorical classification (within normal limits, borderline, or outside normal limits) was also recorded. We defined CON with a glaucoma-like disc as those classified as abnormal or borderline by the glaucoma classification of HRT2 MRA.

### Spectral-domain optical coherence tomography

After pupillary dilation, the optic nerve was imaged using Spectralis OCT (Heidelberg Engineering GmbH, Dossenheim, Germany). For peripapillary retinal nerve fiber layer measurement and peripapillary choroidal thickness, a 3.46-mm-diameter circular scan, centered around the optic disc center, was used. The mean circumpapillary retinal nerve fiber layer (cpRNFL) thickness and circumpapillary choroidal thickness (cpCT) on the OCT image obtained by averaging 50 circular B-scans was used for analysis.

**Figure 1:**
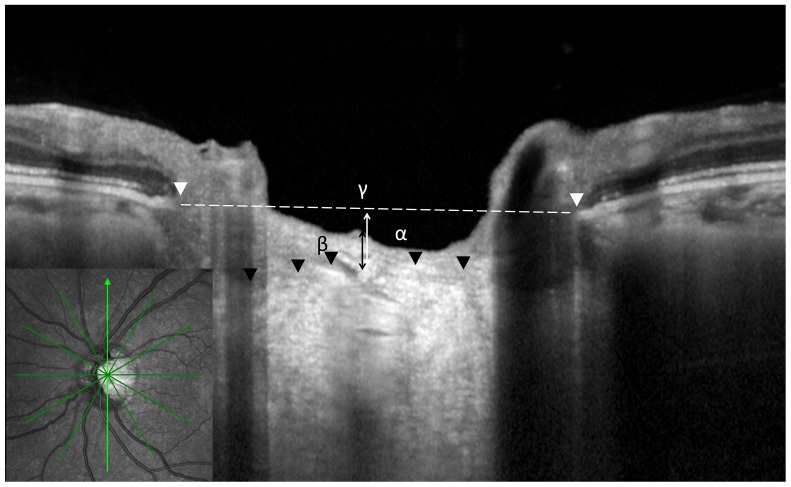
Optic nerve head quantitative parameters measured with enhanced depth imaging optical coherence tomography (OCT). A radial OCT scans at the optic disc were obtained. White arrowheads show Bruch's membrane opening (BMO). Dotted line shows BMO reference plane. Black arrowhead shows the anterior surface of the lamina cribrosa (LC). The distance between BMO reference plane and anterior LC (α), prelamina tissue thickness (β) at the center of BMO reference plane, and the diameters of the BMOs (γ) are also indicated.

The EDI technique was also used for measuring the PLT, diameter of Bruch's membrane opening (BMO), and distance between BMO and the anterior surface of the LC (BMO-anterior LC; Figure 1). The details and advantages of this technology for evaluating the LC have been described previously [14], [20]. A radial scanning pattern centered on the optic disc (24 high-resolution 15° radial scans, each averaged from 50 B-scans with 768 A-scans per B-scan acquired with a scanning speed of 40,000 A-scans per second). The LC appeared as a highly reflective plate-like structure in B-scan images. The start of the highly reflective region within the ONH was considered the anterior border of the LC [21]. We adjusted the brightness and contrast of the images to identify LC border as precisely as possible. The PLT was defined as the distance between the optic cup surface and the anterior border of the highly reflective region that corresponded to the LC. The BMO was defined as the termination of the Bruch's membrane, and we measured the diameter of BMO [22]. The BMO-anterior LC was defined as the vertical distance between the reference line connecting BMO and the anterior laminar surface. In principle, we measured the PLT, diameter of BMO, and BMO-anterior LC at the midpoints between the BMOs on both vertical and horizontal images. An average value was obtained from two images.

### Statistical analyses

All values are presented as mean ± standard deviation. Differences in parameters between two groups were compared by using the unpaired *t*-test or the Mann-Whitney U test, and differences among the 3 groups were compared by analysis of variance (ANOVA) followed by the Tukey post hoc test. Differences in categorical variables were evaluated by χ^2^ tests. To evaluate the interobserver reproducibility of our measuring method for cpCT, PLT, diameter of BMO, and BMO-anterior LC, all SD-OCT datasets from each group were evaluated by 2 examiners (MH and KM), and the interclass correlation coefficient (ICC) was calculated. The diagnostic accuracy was determined by computing the area under the receiver operating characteristic curve (AUROC), and the sensitivity at the fixed specificity was determined to differentiate between CON eyes with a glaucoma-like disc and eyes with glaucoma [23]. The unpaired *t*-test scores, Mann-Whitney U test, ANOVA, and ICC were calculated using SPSS version 19.0.0 statistical software (IBM Japan, Tokyo, Japan), and the AUROCs were compared using MedCalc version 12 (MedCalc Software, Ostend, Belgium). The cutoff points were calculated with MedCalc version 12 as the points with the best sensitivity-specificity balance. Sensitivity at fixed specificities of 90% and 95% (10% and 5% false positive rate, respectively) and positive and negative likelihood ratios were also calculated. A *P* value <0.05 was considered statistically significant.

## Results

During the enrollment period, 34 subjects with compressive optic neuropathy were included. Furthermore, 63 glaucoma and 60 healthy control subjects were initially included. After age matching, this study included 68 eyes of 68 subjects (34 eyes of 34 age-matched glaucoma subjects and 34 eyes of 34 age-matched normal control subjects).

Of the 34 subjects with CON, 13 had pituitary adenomas, 9 had intracranial meningiomas, 9 had craniopharyngiomas, 2 had intracranial aneurysms, and 1 had a sinus mucocele. The descriptive data of the participants are summarized in Table 1. No significant differences were found with regard to age, refraction, sex, axial length, and intraocular pressure when the CON group was compared to either the glaucoma or healthy group. The MD of the visual field was −18.9±8.1, −17.3±6.4, and −0.6±1.7 in the CON, glaucoma, and healthy groups, respectively. There was no significant difference in the MD between the CON and glaucoma groups (*P* = 0.38), but both groups had a significantly poorer MD than the healthy group (*P*<0.001).

**Table 1:** Baseline Patient Characteristics.

	CON	Glaucoma		normal subjects	
			*P* value*		*P* value†
Number of eyes	34	34	-	34	-
Age (years)	59.2±13.2	59.5±13.5	0.94	59.4±14.6	0.95
Sex (male/female)	12/22	19/15	0.14	14/20	0.80
Spherical equivalent (Diopter)	−1.7±2.5	−2.0±2.3	0.63	−1.1±2.6	0.31
Axial length (mm)	24.0±1.3	24.3±1.2	0.31	24.1±1.2	0.56
Intraocular pressure (mm Hg)	15.8±3.7	16.3±3.7	0.58	16.3±3.5	0.51
Mean deviation (dB)	−18.9±8.1	−17.3±6.4	0.38	−0.6±1.7	<0.001

CON =  compressive optic neuropathy

*comparison between CON and glaucoma

† comparison between CON and normal subjects

**Figure 2:**
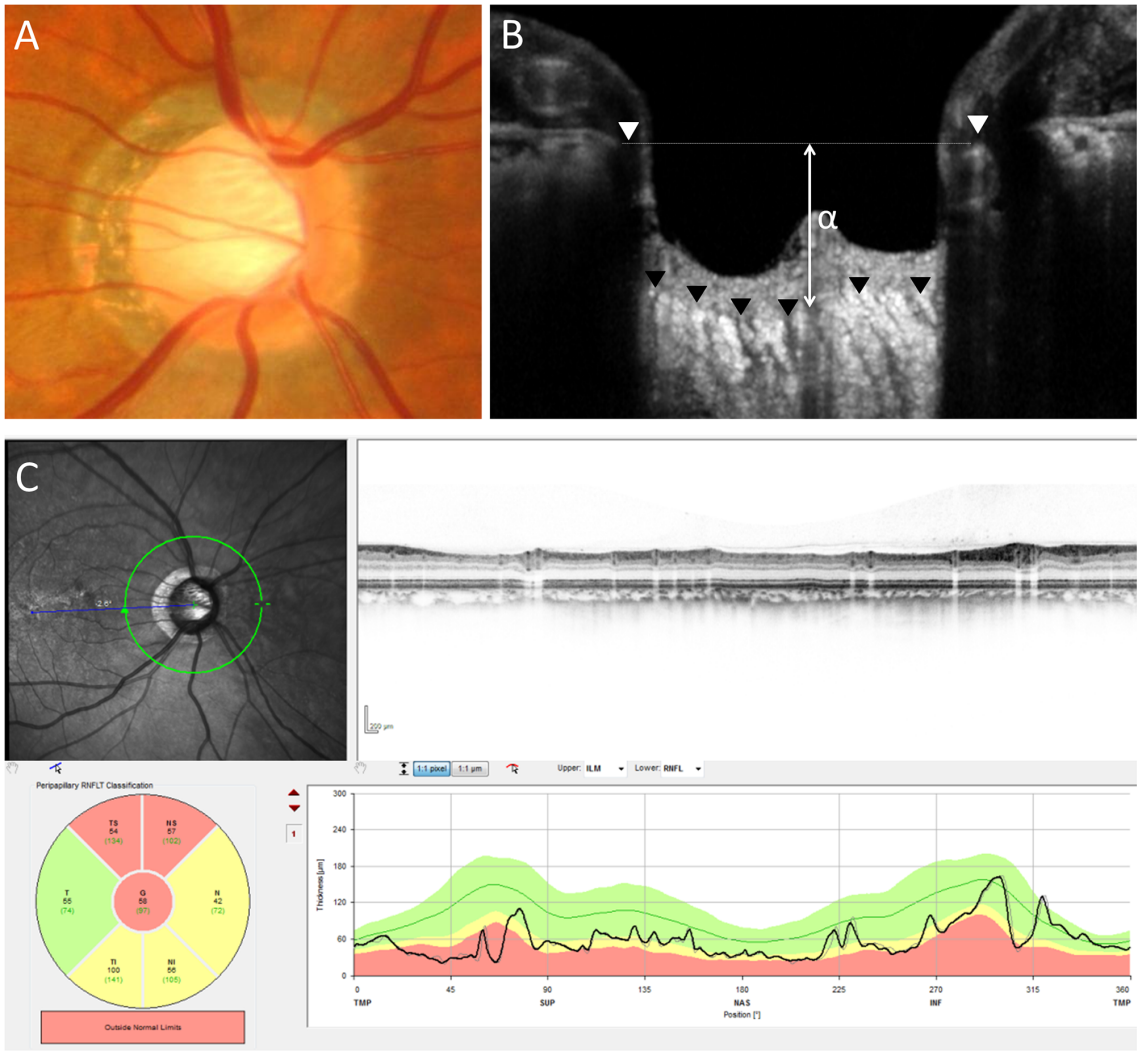
Representative case of eye with glaucoma. A fundus photograph (A) shows a glaucomatous disc with enlarged optic disc cupping and upper rim loss. A vertical OCT scan (B) shows a distance of 406 µm between Bruch's membrane opening reference line and the anterior lamina cribrosa (α). A circular OCT (C) scan shows thinning of the circumpapillary retinal nerve fiber layer (58 µm).

**Figure 3:**
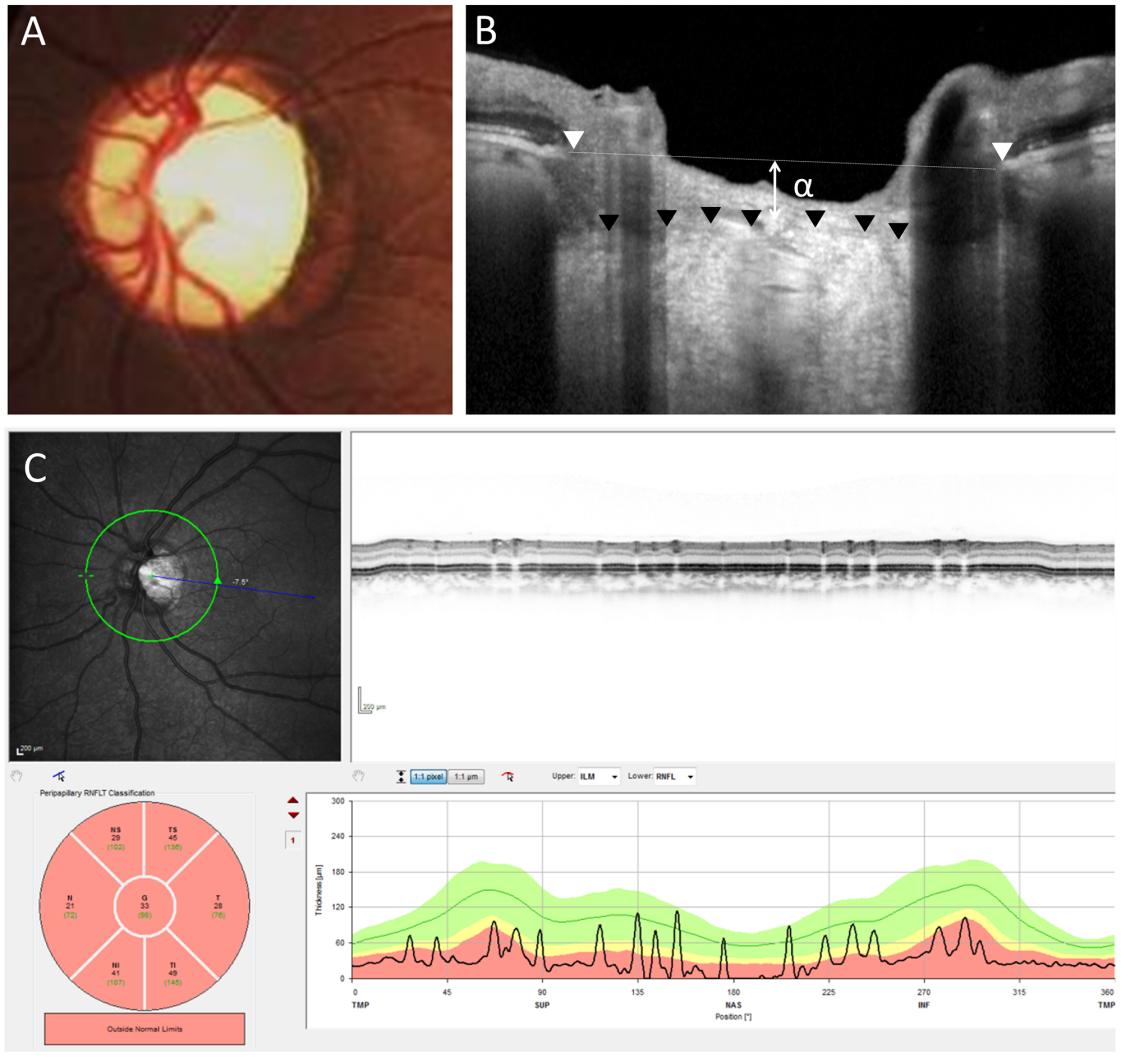
Representative case of eye with compressive optic neuropathy caused by a meningioma. A fundus photograph (A) shows optic nerve atrophy with pallor and enlarged optic disc cupping. A vertical OCT (B) scan shows a distance of 165 µm between Bruch's membrane opening reference line and the anterior lamina cribrosa (α). A circular OCT (C) scan shows severe thinning of the circumpapillary retinal nerve fiber layer (33 µm).

Table 2 shows the comparison of the OCT and HRT parameters of the CON group to the glaucoma and healthy groups. The ICC for cpCT, PLT, the diameter of BMO, and the BMO-anterior LC were 0.92, 0.92, 0.96, and 0.96, respectively; these findings indicate good reliability in the measurement of these parameters. There were no significant differences in the diameter of the BMO and disc size in HRT measurements among the 3 groups. The mean cpRNFL of the CON group was significantly thinner than that of the healthy group and that of the glaucoma group (*P*<0.001 and *P* = 0.03, respectively). The C/D area ratio of the glaucoma group was significantly greater than that of the CON group, and the ratio of the CON group was significantly greater compared to that of the healthy group (*P*<0.001 and *P* = 0.006, respectively). The mean PLT of the CON group was thinner than that of normal subjects but was almost the same as that of the glaucoma group (*P* = 0.001 and *P* = 0.75, respectively). Among HRT parameters, the mean and maximum cup depths of glaucoma were significantly greater than those in the CON group (*P*<0.001 and *P*<0.001, respectively). In EDI-OCT, the BMO-anterior LC of the glaucoma group was also significantly greater than that of the CON group, and the BMO-anterior LC of the CON group was similar to that of the healthy group (*P*<0.001 and *P* = 0.47, respectively) (Figures 2 and 3). In the CON group, the mean cpCT was 169.8±52.6 µm, which was similar to that of the healthy group (171.1±44.3 µm, *P* = 0.92). The mean cpCT in the glaucoma group was 157.2±48.9 µm, and there was no significant difference between the glaucoma group and the CON group (*P* = 0.32).

**Table 2:** Comparison of OCT and HRT parameters among CON, glaucoma, and normal subjects.

	CON (N = 34)	glaucoma (N = 34)		normal subjects (N = 34)	
			*P* value*		*P* value†
cpRNFL (µm)	51.3±14.5	58.6±12.5	0.03	97.2±8.8	<0.001
PLT (µm)	116.3±59.6	111.7±59.8	0.75	183.5±97.7	0.001
Width of BMO (µm)	1659.2±155.7	1655.2±168.1	0.92	1617.5±217.6	0.37
cpCT (µm)	169.8±52.6	157.2±48.9	0.32	171.1±44.3	0.92
Disc size (mm)	2.03±0.53	2.21±0.46	0.16	2.04±0.57	0.96
BMO-anterior LC (µm)	324.0±114.2	508.4±157.8	<0.001	348.2±156.8	0.47
C/D area ratio	0.35±0.15	0.52±0.17	<0.001	0.25±0.14	0.006
Mean cupping depth (mm)	0.19±0.07	0.33±0.11	<0.001	0.22±0.08	0.16
Max cupping depth (mm)	0.46±0.14	0.74±0.21	<0.001	0.58±0.16	0.002

CON =  compressive optic neuropathy; OCT =  optical coherence tomography; HRT =  Heidelberg Retina Tomograph; cpRNFL  =  circumpapillary retinal nerve fiber layer; PLT  =  prelamina tissue thickness; BMO =  Bruch's membrane opening; cpCT =  circumpapillary choroidal thickness; BMO-anterior LC =  distance between BMO and anterior surface of lamina cribrosa.

*comparison between CON and glaucoma.

† comparison between CON and normal subjects.

Of 34 eyes with CON, 17 eyes (50%) presented with a cup-to-disc ratio of 0.8 or greater on stereoscopic photographs. Based on the glaucoma classification of HRT2 MRA, 15 (44.1%) of 34 eyes with CON were classified as abnormal or borderline (defined as CON with a glaucoma-like disc). Table 3 shows the comparison of OCT and HRT parameters between CON with a glaucoma-like disc by HRT classification and glaucoma. The BMO-anterior LC and the mean and maximum cup depths of CON subjects with a glaucoma-like disc were smaller than those of the glaucoma group (*P* = 0.005, *P* = 0.003, and *P* = 0.001, respectively).

**Table 3:** Comparison of OCT and HRT parameters between eyes with CON with a glaucoma-like disc and eyes with glaucoma.

	CON (N = 15)	glaucoma (N = 34)	*P* value
cpRNFL (µm)	49.0±15.0	58.6±12.5	0.02
PLT (µm)	124.5±70.0	111.7±59.8	0.52
Width of BMO (µm)	1699.0±128.2	1655.1±168.1	0.37
Disc size (mm)	2.17±0.46	2.21±0.46	0.80
BMO-anterior LC (µm)	376.2±102.6	508.4±157.8	0.005
C/D area ratio	0.46±0.11	0.52±0.17	0.16
Mean cupping depth (mm)	0.23±0.07	0.33±0.11	0.003
Max cupping depth (mm)	0.52±0.14	0.74±0.21	0.001

CON =  compressive optic neuropathy; OCT =  optical coherence tomography; HRT =  Heidelberg Retina Tomograph; BMO-anterior LC =  distance between Bruch's membrane opening and anterior surface of lamina cribrosa.

To differentiate between eyes with CON with a glaucoma-like disc and eyes with glaucoma, the AUROCs, best sensitivity-specificity balance, and sensitivity at 90% and 95% specificities were calculated for BMO-anterior LC, mean cup depth, and maximum cup depth (Table 4). Overall, the AUROCs of the mean cup depth and maximum cup depth were above 0.800.

**Table 4:** Diagnostic accuracy determined by computing the AUROCs, best sensitivity-specificity balance, likelihood ratios, and sensitivity at the fixed specificities for the optic nerve head parameters of OCT and HRT to discriminate between CON eyes with a glaucoma-like disc and glaucoma eyes.

OCT and HRT parameters	AUROC	95% CI	AUROCs *P* Value	Cutoff Point	Sensitivity (%)	Specificity (%)	+LR	−LR	Sensitivity	
									Specificity 90%	Specificity 95%
BMO-anterior LC	0.754	0.610–0.866	0.0009	395 µm	73.33	79.41	3.56	0.34	33.33	13.33
Mean cupping depth	0.803	0.662–0.903	<0.0001	0.27 mm	85.71	67.65	2.65	0.21	41.43	14.29
Maximum cupping depth	0.829	0.692–0.922	<0.0001	0.6 mm	85.71	73.53	3.24	0.19	57.14	28.57

AUROC =  area under the receiver operating characteristic curve; OCT =  optical coherence tomography; HRT  =  Heidelberg Retina Tomography; CI =  confidence interval; +LR =  positive likelihood ratio; −LR =  negative likelihood ratio; BMO-anterior LC =  distance between Bruch's membrane opening and anterior surface of lamina cribrosa.

Note: The cutoff points were calculated using the MedCalc software as the points with the best sensitivity-specificity balance. Sensitivities at fixed specificities of 90% and 95% are shown.

## Discussion

The purpose of the current study was to determine whether there are any differences in quantitative parameters of the ONH between eyes with CON and eyes with glaucoma and to identify parameters useful in detecting intracranial lesions among eyes with enlarged optic disc cupping. Compared to eyes with glaucoma, eyes with CON had a shallower cup depth. Additionally, 50% of eyes with CON presented with enlargement of the optic cup on fundus photographs, and 44.1% were judged to have a glaucoma-like disc by HRT classification. For distinguishing CON with a glaucoma-like disc from glaucoma, ONH parameters, specifically a shallow maximum cup depth, increase the likelihood of identifying an intracranial mass lesion.

This study elucidated the difference in ONH deeper structures between glaucoma and CON. Our results indicate that different mechanisms may result in disc cup enlargement. Glaucomatous cupping has been histologically shown to result from the loss of both axons and astroglia in the optic disc [24], [25] and posterior LC displacement and thinning of the LC [11], [26]–[28]. Compression of the afferent visual pathway also causes enlargement of the optic nerve cup. Portney and associates conducted a pathological study in a case of disc cupping associated with compression of the optic nerve and showed loss of axons and reported that glial tissue within the optic nerve head would account for the cupping [7]. However, whether axonal loss alone is a sufficient explanation for cupping in CON has been unknown.

This study used a new EDI-OCT technique, which has been shown to reliably capture the LC and the deep structures of the ONH in normal eyes and eyes with glaucoma. Our study showed that eyes with CON had a smaller LC depth than eyes with glaucoma and that LC depth of the CON group was similar to that of the healthy group. Previous studies have reported that eyes with glaucoma presented with posterior LC displacement and a thinner LC than normal eyes [14], [15], [17], [29]. Our results indicated that retrograde axonal degeneration caused by optic nerve compression does not accompany laminar remodeling, which is unlike that observed in glaucomatous optic neuropathy. This result was consistent with the hypothesis that the pathogenesis of optic nerve degeneration in glaucomatous optic neuropathy is located at the LC, while the pathogenesis of other optic neuropathies is not [11], [30]–[32].

Our study revealed that disc cupping depth and LC depth are useful diagnostic parameters in detecting CON with a glaucoma-like disc. Compressive anterior visual pathway lesions can mimic both glaucomatous disc cupping [3] and field defects [33], and distinguishing them from glaucoma is often difficult. Ahmed and associates state that it would be difficult for a normal clinician rather than a neuro-ophthalmologist to diagnose most forms of optic neuropathy and, thus, advocates routine neuroimaging [34]. However, routine neuroimaging for subjects suspected to have glaucoma has a low sensitivity for detecting intracranial lesions [35], [36]. To improve the cost-to-benefit ratio of neuroimaging, a more selective approach is needed. Among quantitative parameters investigated in this study, maximum cupping depth has good specificity and sensitivity. A detailed assessment of the ONH provides the information necessary to distinguish cupping caused by glaucoma versus compression.

The LC depth in the patients with CON was smaller than that in the glaucoma group, but this is not a useful diagnostic parameter in detecting CON with glaucoma-like discs. In our study, we measured the LC depth as the distance between the BMO and the anterior surface of the LC, as has been previously reported [18]. However, the use of the BMO as a reference plane for deep optic nerve structures may be influenced by the choroidal thickness [37]. In fact, the peripapillary choroidal thickness has been reported to be thinner in patients with normal tension glaucoma compared to normal subjects [38]. Although there was no significant difference in the cpCT between the CON and glaucoma groups in the present study, the difference of choroidal thickness could underestimate the differences between CON and glaucoma.

This study has several limitations. In addition to the relatively small sample size, poor fixation of patients could impair the reliability of visual field testing because some CON and glaucoma patients had poor visual acuity. Additionally, the results of visual field testing do not represent all visual fields. In the present study, all patients with glaucoma and CON were Japanese and of Asian ancestry, and high prevalence of glaucoma is reported among Japanese individuals. Differences in glaucoma prevalence according to race might be a factor in differentiating between the two diseases [39]. Diagnostic tests are indicated for use in patients with suspected disease and not in patients with confirmed CON or glaucoma; however, we compared only patients who had already been diagnosed with CON or glaucoma. A further prospective study should be performed in a larger population. As for the HRT measurement, the cup size and rim can be artifactually smaller using the HRT because the reference plane is selected to be temporal, in which the papillo-maculo bundle is the first to be affected in eyes with CON [40]. In fact, the incidence of a glaucoma-like disc in eyes with CON was smaller with evaluation by HRT than that by stereo disc photography in our study. In addition, although as many as one-half of patients with CON presented with a glaucoma-like disc, we could not exclude the possibility that some of them may have had an underlying glaucomatous process. Despite these limitations, quantitative analysis of ONH morphology provides the necessary evidence to distinguish cupping caused by intracranial lesions from that caused by glaucoma.

## Supporting Information

File S1 **Specific data of each group.** Detail data of group1 (glaucoma), group2 (normal), group3 (compressive optic neuropathy), and group4 (compressive optic neuropathy accompanying with glaucomatous disc).
(XLSX)
